# Comparison Study of the Histomorphometric Results after Socket Preservation with PRF and Allograft Used for Socket Preservation—Randomized Controlled Trials

**DOI:** 10.3390/ijerph18147451

**Published:** 2021-07-13

**Authors:** Vasilena Ivanova, Ivan Chenchev, Stefan Zlatev, Eitan Mijiritsky

**Affiliations:** 1 Oral Surgery Department, Faculty of Dental Medicine, Medical University of Plovdiv, 4000 Plovdiv, Bulgaria; 2 Center of Dental Implantology Research Institute, Medical University of Plovdiv, 4000 Plovdiv, Bulgaria; ivan.chenchev@mu-plovdiv.bg; 3 CAD/CAM Center of Dental Medicine, Research Institute at the Medical University of Plovdiv, 4000 Plovdiv, Bulgaria; stefan.zlatev@mu-plovdiv.bg; 4 Head and Neck Maxillofacial Surgery, Tel-Aviv Sourasky Medical Center, Department of Otoryngology, Sackler Faculty of Medicine, Tel-Aviv University, Tel-Aviv 699350, Israel; mijiritsky@bezeqint.net

**Keywords:** dental implants, allograft, PRF, vital bone, histology, bone biopsy

## Abstract

The aim of the present clinical study was to assess and compare the histomorphometric results and efficacy of freeze-dried bone allograft (FDBA) in combination with platelet-rich fibrin (PRF), and PRF as a sole grafting material for socket preservation. Ninety patients in need of tooth extraction and implant restoration were included in this study. The participants were randomly divided into three groups based on post-extraction clinical protocol: socket preservation procedure with allograft in combination with a PRF membrane (PRFm), PRF as a sole grafting material, and a control group. A total of 90 implants were placed four months post-extraction. During the surgical re-entry a bone biopsy was harvested with a trephine drill. Histological samples were prepared and analyzed for percentage vital bone and connective tissue. One-way ANOVA with Bonferroni post-hoc analysis were used to assess the results. Both test groups revealed a significantly higher percentage of vital bone formation compared to the control group. No statistically significant differences regarding vital bone formation and connective tissue quantity between the tested groups were observed (FDBA + PRFm: 3.29 ± 13.03%; and PRF: 60.79 ± 9.72%). From a clinical and histological point of view, both materials in the test groups are suitable for the filling of post-extraction sockets without bone defects. Both of the tested groups revealed a significantly higher percentage of vital bone formation compared to the control group.

## 1. Introduction

After tooth extraction the dimensions of the alveolar bone are subjected to resorption and remodeling processes. These changes occur after tooth loss, given that the alveolar process is tooth dependent [1]. Progressive bone resorption causes difficulties in the placement of conventional prostheses and implant-supported prostheses, and in many cases implant placement becomes a challenge. Studies have reported that after tooth extraction bone resorption is most active in the first year, and is most pronounced during the first three months [2]. Around 50% of the alveolar bone width is lost within 12 months of extraction, 30% of which occurs within the first 12 weeks [3]. Studies have shown decreases in bone width and height of 2.6–4.5 mm and 0.4–3.9 mm, respectively, after tooth extraction without socket preservation [4]. Six months after tooth extraction, the width and height of the alveolar bone are reduced by 3.8 and 1.24 mm, respectively [5]. Some studies have reported that post-extraction alveolar resorption of the upper or lower alveolar ridge is significantly more pronounced in the area of the buccal bone plate compared to the lingual/palatal [6]. Araujo et al. [7] reported that eight weeks after tooth extraction the buccal bone plate is positioned 2 mm apically from the lingual.

According to the Osteology Consensus Report of 2012, based on the systematic review by Vignoletti et al. 2012 [8], performing a socket preservation procedure allows the volume of the existing soft and hard tissue to be preserved. The duration of time between the ARP measure and the implant placement must be considered an important factor [9]. Socket preservation procedures enhance functional and aesthetic outcomes, thus avoiding subsequent difficulties in prosthetic rehabilitation and the placement of dental implants in the area [10].

Numerous surgical techniques and grafting materials are used for socket preservation procedures. The materials vary according to their composition, bio-mechanism, and source. The ideal bone graft material should be biologically inert, biodegradable, easy to apply, mechanically resistant, and widely available [11]. One of the most important factors is the ability of the material to be resorbed and substituted by newly formed bone [12].

PRF (platelet-rich fibrin) is a second-generation platelet concentrate obtained after centrifugation of the patient’s own blood. The protocol was first presented in France by Choukroun et al. [13]. A fibrin network is obtained by centrifugation of blood alone and comprises of high amounts of platelets and leukocytes [14]. The collected clot (or biomaterial) is stable, elastic, adhesive, and flexible. It can be cut or adapted to various anatomical defects and used in combination with bone replacement materials as a sole graft material or in the form of a fibrin membrane. PRF has hemostatic, angiogenic, osteogenic, anti-inflammatory, antimicrobial, analgesic, and healing properties [15].

Autogenous bone is considered to be the “golden standard” among bone grafts, as it meets all mechanical and biological criteria for bone replacement material and shows the most successful and predictable results in bone regeneration [16]. However, autografts have a lot of disadvantages. Allografts overcome the need for a donor region and the limits of harvestable quantity [17].

The aim of the present clinical study was to assess and compare the histomorphometric results and efficacy of a freeze-dried bone allograft in combination with PRF, and PRF as a sole grafting material used for socket preservation.

## 2. Materials and Methods

### 2.1. Study Design

**Figure 1:**
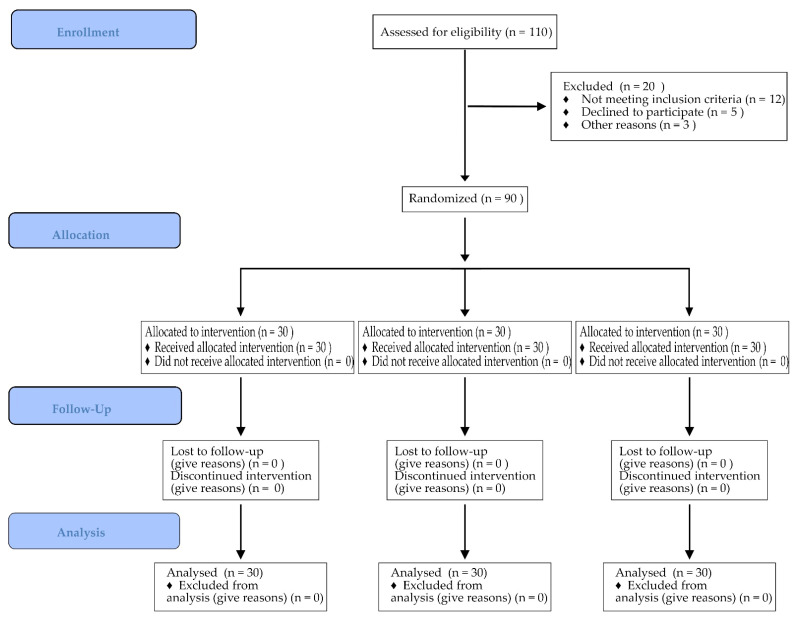
CONSORT flowchart showing the study design and stages–Enrollment, Allocation, Follow-Up, and Analysis.

The present clinical study was performed according to the ethical principles outlined in the declaration of Helsinki. The ethical committee of the Medical university of Plovdiv, Bulgaria approved the study protocol (ethic code: P-2230/26.04.2018). The CONSORT flowchart illustrating the study design and the number of screened and excluded patients is depicted in Figure 1.

Patients were thoroughly informed about the necessity of the procedure, the benefits, and the possible complications. All of them signed a written informed consent before participating in the clinical study. The information regarding the patients was collected and archived in the form of an electronic card accessible for all members of the treatment team.

Ninety patients were enrolled in the randomized clinical study. The relative share of men and women in the study sample was equal, 45 men (50%) and 45 women (50%). The mean age of the patients was 41.60 ± 10.95 years, the youngest being 19 and the oldest 66 years old.

The inclusion criteria were as follows: presence of tooth with indication for extraction (Fractura coronae et radix dentis, Destrucio coronae et radix dentis, Resorbcio radix dentis (interna et externa), Radix dentis; Periodontitis chronica), presence of adjacent teeth, ≥18 years of age, ASA (Physical Status Classification System, American Society of Anesthesiologist) I (normal healthy patient) or II (patient with mild systemic disease), good oral hygiene. The exclusion criteria included: ASA III or IV patients, uncontrolled diabetes, smokers (≥than 10 cigarettes/day), use of immunosuppressing medication, use of anticoagulants, adjacent tooth extractions, or a diffuse infectious process next to the site to be intervened.

Patients Were Divided into Three Groups:

(1) Test group—BoneAlbumin (cortico-cancellous, freeze-dried, serum-albumin impregnated, and 2nd freeze-dried; BoneAlbuminTM, OrthoSera Dental, Hungary) + PRF membrane;
(2) Test group—PRF as a sole grafting material;
(3) Control group—no biomaterial.

Each patient was randomly assigned to one of the two test groups or to the control group—“toss a coin” simple randomization was performed. The allocation of the groups was hidden from the surgeon until immediately prior to the intervention. Due to the nature of the study, neither the surgeon nor the subjects could be blinded to group allocation, but all outcome assessors were masked.

Baseline data and pre-surgical treatment:

At the enrollment visit all demographic data were collected and recorded as well as the dental and medical histories of the patients. All patients underwent a meticulous oral hygiene routine including periodontal treatment before study initiation if indicated.

The baseline data were collected before tooth extraction and included bleeding on probing (BOP), probing pocket depth (PPD), gingival recession (REC) measured on the teeth adjacent to the extraction site, as well as full mouth plaque scores (FMPSs) of less than 30%.

### 2.2. Radiographic Evaluation

Segmental/panoramic radiography was performed at the first patient examination in order to make an accurate diagnosis and determine the need for extraction of the affected tooth. Four months after the socket preservation procedure a CBCT was assigned (Planmeca Romexis Viewer 4.4.3, Planmeca, Helsinki, Finland) for the purpose of implant planning. A control X-ray was performed after implant placement.

### 2.3. Socket Preservation Procedure

**Figure 2:**
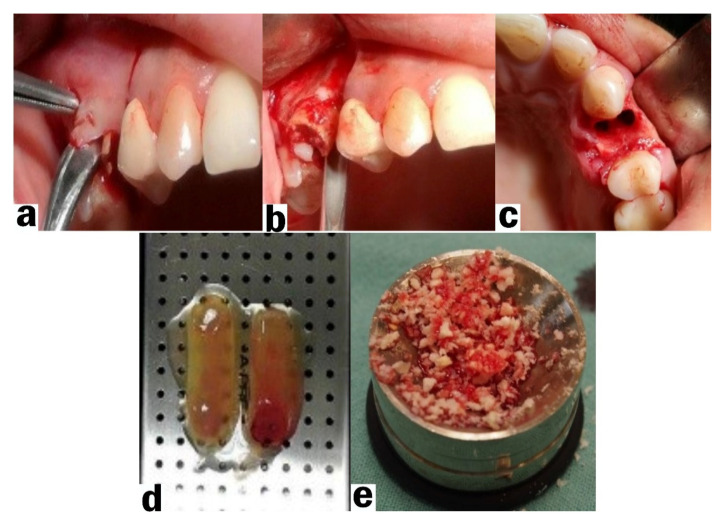
Socket preservation—tooth extraction protocol: (**a**) elevation of a mucoperiosteal flap; (**b**) tooth extraction; (**c**) post-extraction socket and grafting materials used: (**d**) PRF (**e**) Bone graft containing FDBA mixed with blood.

After administering adequate local anesthesia (articaine 4% with epinephrine 1:100.000, 3M ESPE, Seefeld, Germany) an intrasulcular incision around the tooth to be extracted was performed, followed by two releasing incisions reaching the mesial/distal papilla of the adjacent teeth. A trapezoidal mucoperiosteal flap was carefully reflected with a periosteal elevator (Figure 2a). On the palatal side an envelope flap was reflected without additional vertical incisions. Periosteal slitting on the buccal side was performed at the base of the flap. The mucoperiosteal flap was enlarged in order to facilitate coverage of the post-extraction socket without tension. The tooth was extracted with minor surgical trauma with the assistance of periotomes or root sectioning if needed (Figure 2b). The post-extraction socket was carefully debrided and rinsed with sterile saline and 3% hydrogen peroxide. The bone graft material was mixed with several drops of the plasma obtained from the PRF (Figure 2c,d). The post-extraction sockets from the first test group were filled with an allograft to the edge of the bone walls using suitable tools. A platelet-rich fibrin membrane (mPRF) was placed on top of the bone graft. In the second test group, the post-extraction socket was filled with platelet-rich fibrin (PRF) as sole grafting material. The third group of the clinical study (control group) consisted of post-extraction sockets filled only with the blood clot.

The mucoperiosteal flap was adapted in order to cover the post-extraction socket without tension. The flap was sutured with resorbable thread 4/0. They were removed after 7–10 days.

### 2.4. Bone Harvesting and Implant Placement Procedure

**Figure 3:**
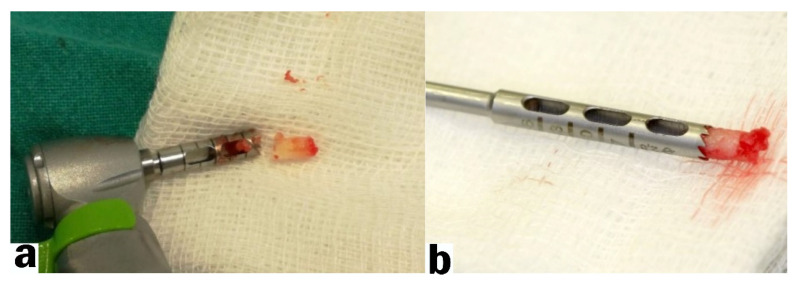
Bone biopsy: (**a**) harvested bone biopsy; (**b**) bone biopsy in a trephine drill.

Four months after the socket preservation procedure a second surgical manipulation was performed. The surgical reentry began with administering adequate local anesthesia. Using a scalpel (blade # 15c), a horizontal incision was made at the site of the previously extracted tooth, followed by two vertical incisions. A biopsy was taken from the exposed bone using a 3 mm trephine burr (Figure 3a,b).

After the bone samples were collected, the osteotomy site was prepared according to the recommendations of the implant system manufacturer (AB Dental Implants, Ashdod, Israel). After the implant placement procedure, a 4/0 resorbable suture was used to close the surgical site (horizontal mattress sutures).

### 2.5. Biopsy Preparation and Examination

**Figure 4:**
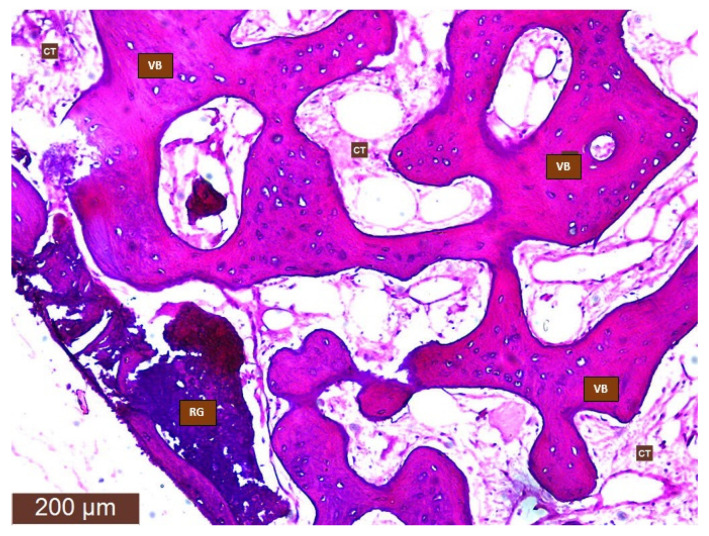
Histological preparation of post-extraction socket four months after tooth extraction. (VB: vital bone; CT: connective tissue; RG: residual graft).

The bone samples were removed from the trephine burr and stored in a small jar with a 10% formalin solution for 12–24 h. The bone fragments were decalcified (decalcifying solution: EDTA; microwave oven: 8 cycles of 10 s at 41–43 °C for 20 min, after each cycle of the microwave oven the decalcifying solution was changed). In the next step (paraffin incorporation), the material was dehydrated with an alcohol series: 70% ethyl alcohol, 95% ethyl alcohol, 99% ethyl alcohol and clarified with xylene. The bone fragments were embedded in a paraffin block, cut into slices (3–4 microns), and stained with Hematoxylin–Eosin (Figure 4).

**Figure 5:**
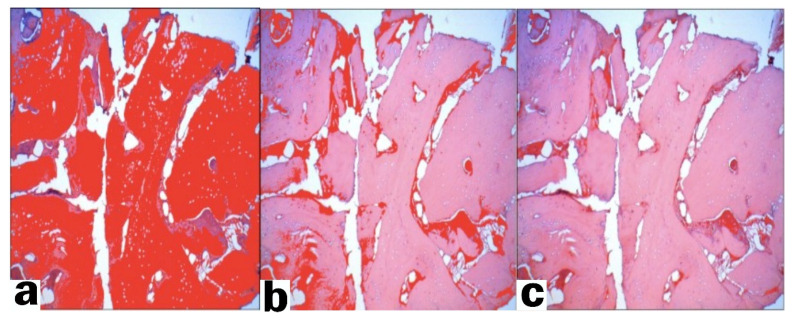
Digital pictures of the microscopic samples: (**a**) newly formed bone area; (**b**) connective tissue area; (**c**) residual graft particles area.

Digital pictures of the microscopic samples were taken and imported into a free image processing program (Fiji/ImageJ) [18]. In the software the percentages of newly formed bone, connective tissue, residual bone graft, and immature bone were calculated (Figure 5a–c). The latter were measured in pixels and converted to percentages. Measurements were performed by two independent researchers.

### 2.6. Postoperative Care

Post-operatively all patients were prescribed antibiotic therapy for five days (Amoxicillin 1 g, every 8 h) and non-steroid anti-inflammatory drugs (Nimesulide 2 × 100 mg) for three days. Patients were instructed to avoid mechanical control of the plaque for 2 weeks in the area of the surgical field. It was recommended that cold compresses should be applied in the area of surgery during the first 48 h and that liquid-nutritious food should be consumed during the first week of recovery. A check-up was performed on the first and the seventh day after surgery in order to control the healing process, the presence of inflammation in the surrounding tissues, or the exposure of a bone graft material. Stitches were removed after ten days.

### 2.7. Outcomes

The clinical study evaluated the quality of the developed tissue measured in percentages: (1) vital bone formation, (2) connective tissue, and (3) residual graft particles and immature bone.

### 2.8. Statistical Methods

The object of the present study was to determine the existence of a relationship between the material used for socket preservation and the newly formed bone found in the histological samples. For this purpose, the percentage of vital bone in the three experimental groups was compared by one-way ANOVA analysis and post-hoc pairwise analysis using the Bonferroni test. The results are presented as the mean ± SD, minimum and maximum value with 95% confidence interval. Statistical significance was reported at a tolerable error level of alpha (alpha) = 0.05 (*p* < 0.5).

Microsoft excel (version 2016) was used for all statistical procedures. Descriptive statistics and graphical analyses were used to characterize the study sample and visualize the results. One-way ANOVA with a Bonferroni post-hoc analysis were used to assess the differences between the three study groups. The results are presented as the mean ± SD, minimum and maximum value with 95 % confidence interval. Statistical significance was reported at a tolerable error level of alpha (alpha) = 0.05 (*p* < 0.5).

## 3. Results

### 3.1. Clinical Results

Ninety patients were enrolled and assigned to one of the three study groups. Equal numbers of patients *(n* = 30) were allocated to each group using a simple randomization procedure to ensure similarity between the experimental groups.

**Figure 6:**
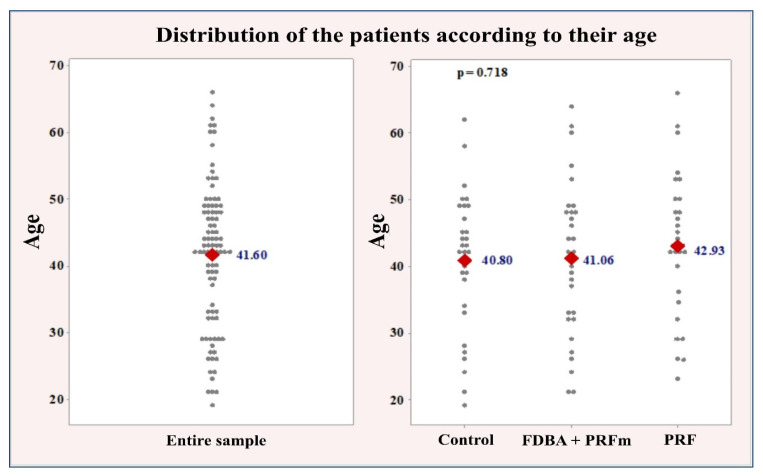
Distribution of the patients according to their age for the entire sample (**left**), and groupwise (**right**).

Groupwise comparisons of participants’ age were performed using one-way ANOVA analysis, whereas sex distribution was assessed with the chi-square test. The mean age of the study sample was 41.60 ± 10.95 years, with the youngest participant 19 and oldest 66 years old. The experimental groups were with similar age distribution and average age, with no statistically significant differences (*p* = 0.718): control: 40.80 ± 10.50 years; bone: 41.06 ± 11.68 years; PRF: 42.93 ± 10.89 years (Figure 6).

The relative share of men and women within the sample is equal, with 45 men (50%) and 45 women (50%). A similar distribution by gender, with no significant difference was found in the study groups (*p* = 0.875): bone: 53% female, 47% male; PRF: 50% female, 50% male; control group: 47% female, 53% male.

**Figure 7:**
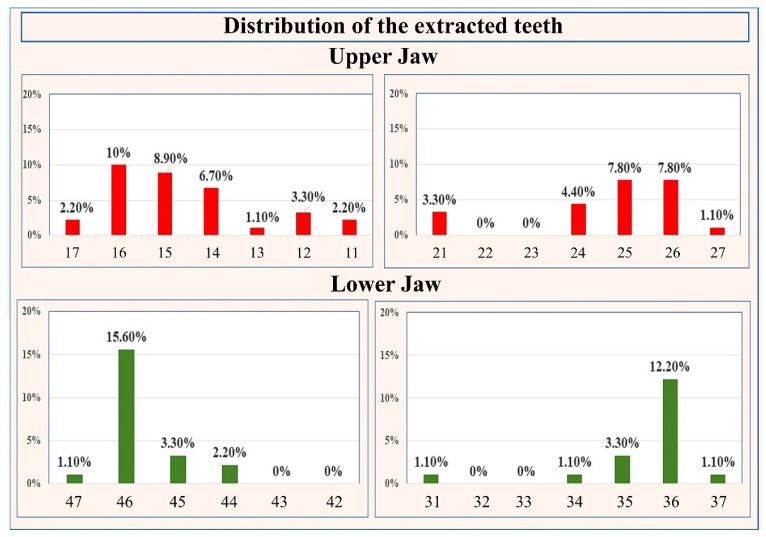
Distribution of the extracted teeth.

The number and percentages of extracted teeth in the upper and lower jaw are summarized in Figure 7. In the upper jaw 53 teeth were extracted, accounting for 58.90% of the entire sample. In the mandible 37 teeth were extracted, constituting 41.10% of the sample. The difference in the relative proportion of extracted teeth between the upper and lower jaw was statistically significant, *p* = 0.025. In the upper jaw, the largest numbers of extracted teeth were the teeth numbered as follows (FDI notation): 16 (*n* = 9; 10%), 15 (*n* = 8; 8.90%), 25 (*n* = 7; 7.80%), and 26 (*n* = 7; 7.80%). In the mandible, the highest numbers of extracted teeth were 46 (*n* = 14; 15.60%) and 36 (*n* = 11; 12.20%).

No complications during the healing period were recorded in any of the participants. Four months after the procedure a non-inflamed keratinized gingiva was observed at the coronal part of the surgery site.

### 3.2. Histological Results

#### 3.2.1. Vital Bone Formation

**Figure 8:**
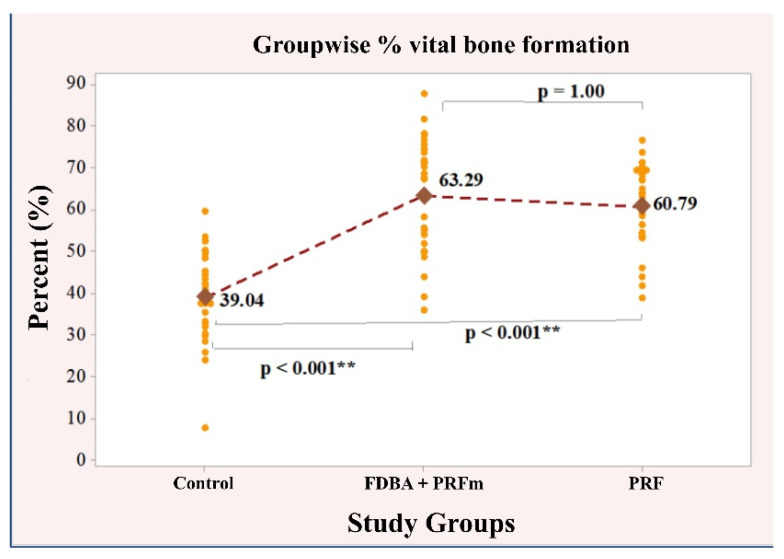
Individual and mean arithmetic values of % vital bone. ** denotes significant difference between groups.

A significant correlation was found between the material used for preservation and the measured values of newly formed bone (ANOVA, *p* < 0.001). More specific information about this dependency was provided by pairwise comparisons (Bonferroni). In terms of arithmetic mean values, the lowest percentage of newly formed bone was found in the control group (39.04 ± 10.89%), which was significantly different from the bone group (63.29 ± 13.03%; *p* < 0.001) and the group with PRF (60.79 ± 9.72%; *p* < 0.001). In both of the tested groups the percentage of newly formed bone was very similar, with a value of *p* = 1.00. The results regarding the relationship between the material used for socket preservation and the amount of bone formed are illustrated in Figure 8.

#### 3.2.2. Connective Tissue

**Figure 9:**
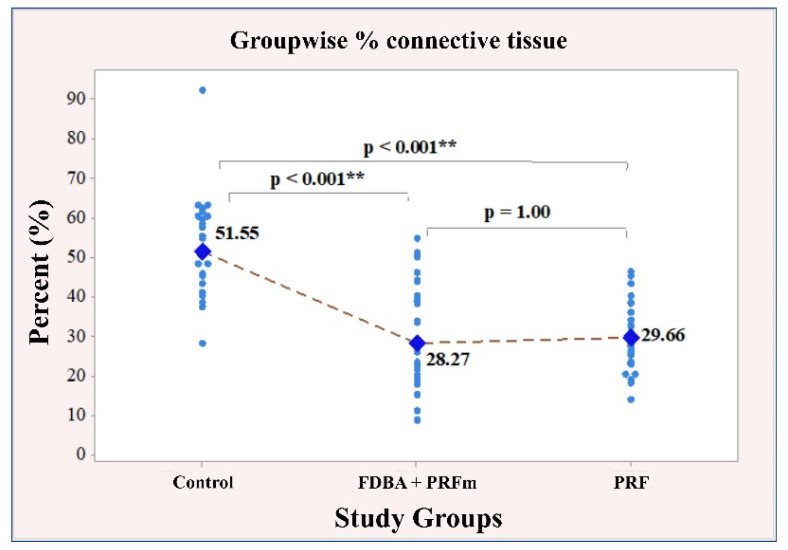
Individual and mean values of % connective tissue. ** denotes significant difference between groups.

A strong, significant correlation emerged between the measured values of connective tissue and the socket preservation material (*p* < 0.001). The subsequent post-hoc analysis specified the results as follows: firstly, the control group of patients had the highest connective tissue value (51.55 ± 12.03), with a significant difference compared to the other two groups: bone (28.27 ± 13.22; *p* < 0.001); PRF (29.66 ± 8.14; *p* < 0.001). Secondly, in the bone and PRF groups, the connective tissue values were very similar, with minimal difference (*p* = 1.00). The results regarding the relationship between the material used for socket preservation and the amount of connective tissue are illustrated in Figure 9.

#### 3.2.3. Residual Bone Graft Particles/Immature Bone

**Figure 10:**
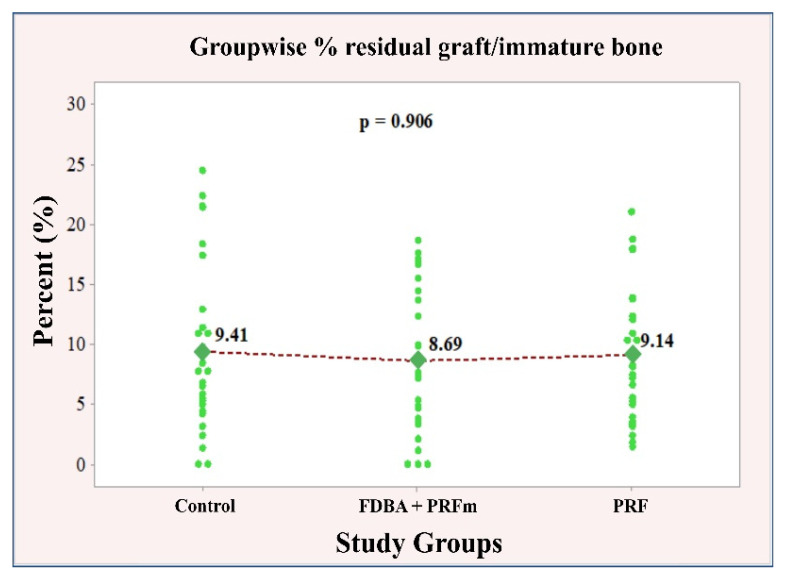
Individual and mean values of % residual graft/immature bone.

Similar values of immature bone/residual graft were identified for all study groups: control 9.41 ± 6.74; bone 8.69 ± 5.99; PRF 9.14 ± 6.25. A statistical analysis of the association between the material used and the amount of immature bone/residual graft showed no dependencies, *p* = 0.906. Therefore, no post-match comparisons in pairs were made in this case (Figure 10).

## 4. Discussion

The healing process of post-extraction socket has been described in numerous clinical and experimental studies [19,20]. Immediately after tooth extraction, the socket is filled with blood from the injured vessels. A blood coagulum is formed consisting of erythrocytes, platelets, and leukocytes covered by a fibrin network. The coagulum plays the role of a physical matrix, guiding the movement of mesenchymal cells and growth factors. Over the next 48–72 h, young granulation tissue composed of polymorphonuclear cells, macrophages, and lymphocytes invades the base and periphery of the socket to the clot and begins to replace it. By the end of the first week, the vascular network is formed, and two weeks after the extraction young connective tissue rich in blood vessels covers the marginal portion of the socket [19]. Trabecular bone begins to form between four and eight weeks after extraction, followed by a bone maturation process. After about 12 weeks of healing, the socket is filled with lamellar bone and bone marrow. A periosteum-like structure is formed from collagen fibers after 120 and 180 days of healing [21]. The healing process of the extraction wound is a collection of both resorption and remodeling of the alveolar bone [22]. This bone resorption leads to reduction of bone and soft tissue volume in both vertical and horizontal directions. Therefore, one of the most important aims in implant dentistry is the development of optimal quality and quantity of bone and soft tissues in which to place dental implants with good long-term functional and aesthetic results.

The most biologically reliable method for bone structure evaluation is the histomorphometric examination of bone biopsies, but it is not routinely applicable in clinical practice [23]. In the study by Zhang et al. [24], 28 patients were divided into two groups: a test group undergoing socket preservation with PRF and a control group. A histologic analysis showed a significantly increased amount of newly formed bone in the test group compared to the control group, suggesting that PRF was able to stimulate bone regeneration. In addition, PRF has also been shown to release autologous growth factors gradually and exert a lasting effect on osteoblast proliferation and differentiation when examined in vitro on rats [25]. The maximum stimulating effect of PRF occurs on day 14 [26]. These findings suggest that the growth factors contained in PRF may play a role in promoting bone regeneration for a prolonged period. Diss et al. [27] in a one-year prospective study of sinus lift using osteotomes and PRF, demonstrated that the PRF fibrin matrix directly promotes angiogenesis. PRF has an osteoconductive and/or osteoinductive property that stimulates bone regeneration [28,29]. In a study in which PRF was added to lyophilized bone allograft (FDBA), spontaneous bone regeneration was reported to accelerate from 8 weeks to 4 months [30]. In a study by Olgun et al. [31], in which the authors compared histologically the effect of a titanium-treated platelet-rich fibrin (T-PRF) or allograft in the formation of new bone, in the T-PRF group, the process was accelerated by up to 4 months compared to the allograft group. In the presented clinical study the results support the angiogenic, osteogenic, and anti-inflammatory properties of PRF as a sole grafting material.

In their study, Choukroun et al. [27] used FDBA and PRF for lateral sinus lift in order to increase bone volume. The authors split the patients into two groups: a control group undergoing sinus lift with FDBA and a test group undergoing sinus lift with FDBA and PRF. A histomorphometric analysis revealed that the bone structures in the control group (FDBA) and the test group (FDBA + PRF) appeared to be similar. These data correlate with the results of our study (mean amount of newly formed bone after socket preservation with PRF: 60.79%; and mean amount of vital bone formed after socket preservation with allograft (FDBA): 63.29%) The addition of PRF to FDBA enables the graft volume to be increased without compromising the quality of the new bone [27]. The fibrin clot acts as a biological binder between the various elements of the graft and as a matrix that allows neo-angiogenesis, stem cell retention and the migration of osteoprogenitor cells into the center of the graft. This is explained by the fact that PRF provides further revascularization of the bone [26]. According to the literature, there is great variability in the amount of newly formed bone, residual graft material, and connective tissue using various bone substitutes and alveolar preservation techniques [32]. This variability can be affected by a number of factors, some of which include the presence of bone dehiscence or fenestration, traumatic extraction of the tooth, damage to the periodontium before tooth extraction, and incorrect angulation when harvesting the biopsy material [33]. In the present study, all teeth were extracted with minimal trauma and post-extraction sockets were carefully examined for bone fenestration or dehiscence. Furthermore, teeth with acute periodontitis were not included. In addition, only the innermost aspect of bone biopsies was analyzed, representing the outermost region of the alveolar bone.

FDBA is an osteoconductive material that functions as a scaffold to induce new bone formation. [34] FDBA has been proven to be a successful bone replacement material in cases where socket preservation aims to maintain bone volume and form new bone [35]. In this study, a freeze-dried bone allograft (FDBA) was used for socket preservation and a biopsy was taken four months after the procedure.

Wood and Mealey [36] investigated post-extraction sockets that had undergone socket preservation with FDBA. Three months after the procedure, an average of 25% of newly formed bone and 25% of residual bone graft were detected on microscopic preparations. Beck and Mealey [33] examined histological findings 14 weeks after preservation of the socket with mineralized bone allograft. The authors reported a mean of 45.8% of newly formed bone, 14.6% of residual material, and 39.6% of connective tissue/non-mineralized tissue. Fotek et al. [37] reported a range of 27% to 32% of newly formed bone 16 weeks after socket preservation with allograft, with an average of 14% to 15% bone graft residues and 53% to 58% connective tissue.

The quantity of newly formed bone in the present study was higher than in the studies done by the aforementioned authors. Four months after the socket preservation procedure, the average amount of newly formed bone was 63.29%. In another study [38], where FDBA was used as a bone graft material and a biopsy from the site was taken five months after the procedure, the authors reported the following results found on microscopic samples: 68% vital bone, 4% residual bone graft, and 38% connective tissue. Iasella et al. [39] reported an average of 54% of newly formed bone six months after socket preservation with FDBA. The results of the last two studies correlate with the results in our study. In the study by Canullo et al. [40] a morphological analysis and semi-quantitative evaluation showed that both at 4 and 12 months of healing after socket preservation with Mg-e HA, all samples had a normal structure without evident presence of inflammatory infiltrate. One of the limitations of the present study is the absence of a test group, including patients in whom only bone graft material without PRF was used for socket preservation. Therefore, complete evaluation of the effectiveness of the allograft as a sole grafting material is not applicable. Another limitation of the present investigation is the recruitment of teeth on both upper and lower jaw. Thus, the results are not specified to a certain area of the alveolar ridge. It is accepted that the amount of new bone formation depends on timing and that the variability between time points or between the participants in the different studies may explain the variability of the data [36].

## 5. Conclusions

From a clinical and histological point of view both materials in the test groups are suitable for the filling of post-extraction sockets without bone defects. Both of the tested groups revealed a significantly higher percentage of vital bone formation compared to the control group. However, there was no statistical differences between the results regarding vital bone formation and connective tissue quantity in the tested groups.

## Data Availability

The data presented in this study are available on request from the corresponding author.
